# The complete chloroplast genome sequence of *Magnolia coriacea* (Magnoliaceae), a critically endangered endemic to China

**DOI:** 10.1080/23802359.2022.2073833

**Published:** 2022-05-10

**Authors:** Yu Li, Baizhu Li, Zhaofeng Li, Yin Yi, Xiaoxin Tang

**Affiliations:** a School of Life Science, Key Laboratory of Plant Physiology and Developmental Regulation, Guizhou Normal University, Guiyang, China; b School of Life Science, Key Laboratory of National Forestry and Grassland Administration on Biodiversity Conservation in Karst Mountainous Areas of Southwestern China, Guizhou Normal University, Guiyang, China

**Keywords:** Chloroplast genome, endemic species, *Magnolia coriacea*, Magnoliaceae, phylogenetic analysis

## Abstract

*Magnolia coriacea*, Chang et B. L. (Magnoliaceae) is a critically endangered tree, endemic to Yunnan province, China. In this study, the complete chloroplast genome of *M. coriacea* was sequenced and analyzed. The total chloroplast genome size of *M. coriacea* is 160,113 bp, including a pair of inverted repeat regions (IRs, 26,576 bp) separated by a large single-copy (LSC, 88,175 bp) region and a small single-copy region (SSC, 18,786 bp). The complete chloroplast genome contains 86 protein-coding (PCGs), 37 transfer RNA (tRNAs), and 8 ribosomal RNA (rRNAs) genes. The phylogenetic analysis showed that *M. coriacea* is closely related to *M. cathcartii*. This study contributes to the bioinformatics on the evolution, genetic, conservation, and molecular biology for future studies of Magnoliaceae.

*Magnolia* is a genus of the family Magnoliaceae, comprising over 60 species; mainly distributed in tropical, subtropical, and temperate regions of Asia (Zhao et al. 2009). There are 32 species currently recognized in China, mainly distributed in the southwest and southeast regions (Cicuzza et al. 2007). Yunnan Province harbors a high number of Magnoliaceae species, many of which are at risk of extinction due to habitat destruction (Fu and Jin 1992; Cicuzza et al. 2007). Among these species, one of the most threatened is *M. coriacea*, Chang et B. L. Chen 1988, which is endangered by habitat destruction, fragmentation, and degradation while its distribution range is entirely unprotected (Tang et al. 2011). *M. coriacea* was described recently and is endemic to Southeast Yunnan, China (Chen 1988; Liu and Wu 1988). This species was said to be similar to *M. martinii* and bears scented whitish or creamy yellow flowers with 6–7 tepals and anthesis lasts from February to April (Chen 1988; Sun and Yan 2006; Zhao and Sun 2009). The existing population of this species is restricted to limestone outcrops in just a few localities at 1300–1700 m (Cicuzza et al 2007; Zhao and Sun 2009; Zhao et al. 2009; Tang et al. 2011). Most of the individuals in the extant populations normally bear flowers, with estimated fruit set and fertile seed production rates at only 6.7% and 0.2%, respectively (Zhao and Sun 2009). *M. coriacea* has been evaluated as a critically endangered tree based on IUCN criteria (Cicuzza et al. 2007). At present, there are no published DNA sequences of *M. coriacea* in GenBank. Here, we assembled and analyzed the complete chloroplast genome of *M. coriacea* using high-throughput sequencing methods. This study contributes to the bioinformatics on the evolution, genetic, conservation, and molecular biology for future studies of Magnoliaceae (Figure 1).

**Figure 1.**
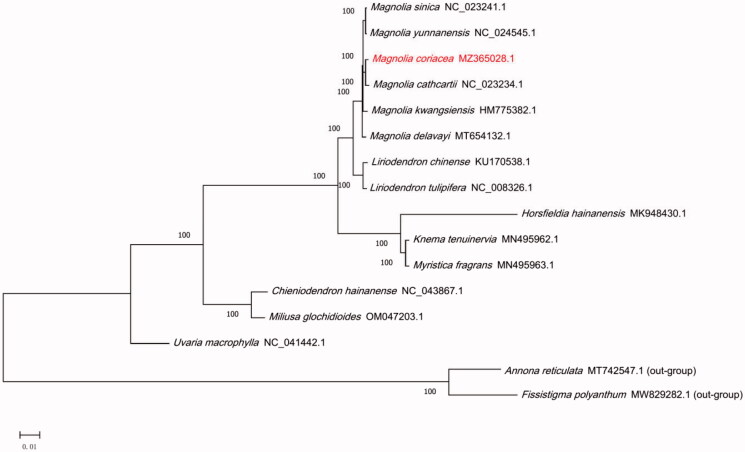
The maximum-likelihood (ML) phylogenetic tree for *M. coriacea* based on 16 chloroplast genome sequences of Magnoliales. Numbers on the nodes are bootstrap values from 1000 replicates.

Fresh young leaves of *M. coriacea* were obtained from Malipo County, Wenshan City, Yunnan Province, China (104° 51′ 14″ E, 23° 10′ 3″ N, 1480 m altitude) and DNA samples were stored at Guizhou Normal University (contact person and email: Baizhu Li, 441248394@qq.com; accession number: HX-001). Total genomic DNA was extracted using the CTAB method (Li et al. 2013). DNA was sent to Shanghai Biozeron Co., Ltd. (Shanghai, China) to construct the DNA library and sequencing using the Illumina HiSeq 6000 sequencing platform. The low quality sequences were removed using Trimmomatic0.39 using the default settings (Bolger et al. 2014). The trimmed reads were selected for the cp genome assembly with the NOVOPlasty4.2 software using the default setting (Dierckxsens et al. 2016). The complete chloroplast genome of *M. coriacea* was annotated by GeSeq (Tillich et al. 2017). The complete cp genome was submitted to GenBank (https://www.ncbi.nlm.nih.gov/search/all/?term=MZ365028; accession number of MZ365028).

The complete cp genome sequence of *M. coriacea* is 160,113 bp and shows a characteristic quadripartite circular structure. The GC content of the complete genome is 39.24%. The large single-copy (LSC) (GC, 37.92%) region is 88,175 bp and the small single-copy (SSC) (GC, 34.26%) region is 18,786 bp. These are separated by a pair of IRa and IRb regions of 26,576 bp. The chloroplast genome of *M. coriacea* has 131 genes in total, including 86 protein-coding, 37 transfer RNA (tRNA), and eight ribosomal RNA (rRNA) genes. Among these, duplication events occurred with 17 genes (ndhB, rpl2, rpl23, rps7, rps12, ycf1, ycf2, trnM-CAU, trnV-GAC, trnI-GAU, trnA-UGC, trnR-ACG, trnN-GUU, rrn16, rrn23, rrn4.5, and rrn5). In addition, 246 simple sequence repeat loci were identified in the cp genome. These were mainly composed of mononucleotide (A, 19.1%; T,26.8%) and trinucleotide (29.67%) repeats by MISA-web (Thiel et al. 2003; Beier et al. 2017). We compared the basic characteristics of the complete chloroplast genome of the genus *M. coriacea* with those of 17 other currently published species of the genus Magnolia, and found that they all have a characteristic quadripartite circular structure, the size (160,113 bp) and GC content (39.24%) of *M. coriacea* were not significantly different from those of the genus Magnolia species (Hall 1999; Li 2021).

In order to further determine the phylogenetic position of *M. coriacea*, 16 cp genome sequences from Magnoliales family were downloaded from GenBank, and two Annonaceae family species were selected as outgroup. Based on MAFFT function under BioAider1.0 software was used for the alignment of cp genome sequences (Zhou et al. 2020). A maximum-likelihood (ML) tree was constructed using PhyML 3.0 online website with the GTR model and 1000 bootstrap replicates (Guindon et al. 2010). The phylogenetic analysis showed that *M. coriacea* was closely related with *M. cathcartii*, and they form a group with other seven species of the genus Magnoliaceae. These data provide important bioinformatic resources for species identification, and phylogenetic relationships within the Magnoliales.

## Data Availability

The genome sequence data that support the findings of this study are openly available in GenBank of NCBI at https://www.ncbi.nlm.nih.gov/search/all/?term=MZ365028 under the accession no. MZ365028. The associated BioProject, SRA, and BioSample numbers are PRJNA774168, SRR16563234, and SAMN22556078, respectively.
